# Downregulation of HOTAIR Expression Mediated Anti-Metastatic Effect of Artesunate on Cervical Cancer by Inhibiting COX-2 Expression

**DOI:** 10.1371/journal.pone.0164838

**Published:** 2016-10-13

**Authors:** Lixin Zhang, Hua Qian, Min Sha, Zhengyun Luan, Mei Lin, Donglan Yuan, Xiaokang Li, Junxing Huang, Lihua Ye

**Affiliations:** 1 Department of Reproductive Medicine, Taizhou People’s Hospital Affiliated of Nantong University of Medicine, Taizhou 225300, China; 2 Department of Gynecology, Taizhou People’s Hospital Affiliated of Nantong University of Medicine, Taizhou 225300, China; 3 Institute of Clinical Medicine, Taizhou People’s Hospital Affiliated of Nantong University of Medicine, Taizhou 225300, China; 4 Institute of Oncology, Taizhou People’s Hospital Affiliated of Nantong University of Medicine, Taizhou 225300, China; Beijing Cancer Hospital, CHINA

## Abstract

Artesunate (ART) has anti-cancer activities for a variety of solid tumors. The aim of this study was to investigate the anti-metastatic effect of ART on cervical cancer cells. *In vivo* anti-metastatic effect of ART was investigated in mice with the lung metastasis model by the subcutaneous injection of ART. The interaction of HOTAIR and COX-2 was measured by RNA immunoprecipitation and RNA pull-down assay. The effect of ART on metastasis of CaSki and Hela cells was evaluated by invasion and migration assay. We found that ART inhibited cervical cancer metastasis and HOTAIR expression. HOTAIR overexpression partially abolished the anti-metastatic effect of ART on cervical cancer cells. In addition, HOTAIR can interact with COX-2 to positively regulate COX-2 expression and catalytic activity. Finally, overexpression of COX-2 reversed the effect of HOTAIR knockdown on Hela cell migration and invasion. Taken together, our data revealed that ART may elicit anti-metastatic effect against cervical cancer by inhibition of HOTAIR expression, which resulted in the decrease of COX-2 expression.

## Introduction

Cervical cancer is a leading cause of cancer-related death among females worldwide, particularly in developing countries [1]. The 5-year survival rate is about 90% if patients were treated in the early stages. Nevertheless, patient outcome is poor when the cancer has metastasized [2]. The traditional strategies for the treatment including surgery, radiotherapy and chemotherapy are not effective to metastatic patients and have severe side effects [3]. Therefore, there is a renewed interest in the use of natural sources to treat cervical cancer.

Artesunate (ART), a common traditional Chinese medicine, has anti-cancer activities for a variety of solid tumors [4–5]. Interestingly, ART has been shown to have a good safety profile exhibiting highly selective anti-tumor actions [6]. The mechanisms of action of ART are involved in the induction of cell cycle arrest and apoptosis of cancer cells, as well as anti-angiogenesis and anti-metastasis [7–8]. Our previous study revealed the molecular mechanism of ART anti-immunosuppressive effect on cervical cancer *in vivo* and *in vitro* [9]. However, the effect and mechanism of ART on metastasis of cervical cancer has not been completely investigated.

The expression of cyclooxygenase (COX)-2 is up-regulated in cervical cancers cells [10]. Overexpression of COX-2 is associated with lymph node metastasis and has been considered a predictor of metastatic potential in cervical cancer [11]. Further study showed that COX-2 and its catalytic product PGE_2_ could induce expression of metalloproteinases (MMP) and vascular endothelial growth factor (VEGF) [12]. In contrast, selective COX-2 inhibitors blocked angiogenesis and suppressed tumor cell invasion [13]. These findings indicated that COX-2 contributes to tumor metastasis and acts as the key molecular to treat cervical cancer. Our previous study demonstrated that ART inhibited COX-2 expression in cervical cancer cells [9]. However, the mechanism by which molecular factors regulate COX-2 expression and activity in cervical cancer cells treated with ART remains unclear.

Long noncoding RNAs (LncRNAs) are longer than 200 nucleotides in length and implicated in a variety of biological processes [14]. Recently, LncRNA HOTAIR (HOX transcript antisense intergenic RNA) has received the most attention in carcinogenesis and metastasis [15]. A meta-analysis revealed that HOTAIR overexpression correlated with lymph node metastasis in many cancers including cervical cancer [16]. HOTAIR knockdown led to a decrease of proliferation, migration, and invasion in cervical cancer cells [17]. In this study, we have found that HOTAIR expression was significantly inhibited in cervical cancer cells induced by ART. We also found that HOTAIR stabilized COX-2 protein. We therefore hypothesized that HOTAIR regulating COX-2 expression might be involved in the effect of ART on cervical cancer. We tested this hypothesis using cervical cancer cells and the mice cervical cancer model.

## Method and Material

### Chemicals and reagents

Dulbecco-modified Eagle medium (DMEM) and Lipofectamine 2000 transfection reagent were obtained from Invitrogen Life Technologies (Grand Island, NY, USA). Fetal bovine serum (FBS) was purchased from GIBCO (Burlington, ON, USA). ART was purchased from Bide Pharmaceutical Corporation (Guangzhou, China). PGE_2_ was purchased from Sigma Aldrich (St Louis, MO, USA). The human PGE_2_ ELISA kit was obtained from Uscn Life Science Inc (Wuhan, China). Antibodies against COX-2 and β-Actin were obtained from Santa Cruz Biotechnology (Santa Cruz, CA, USA). The Detergent Compatible (DC) Protein Assay kit was purchased from Bio-Rad Laboratories (Hercules, CA, USA).

### Cell culture

Human cervical cancer cell lines CaSki and HeLa were obtained from the American Type Culture Collection (Manassas, VA). All cell lines used are genotyped and checked for mycoplasma contamination on a regular schedule. Cells were grown in DMEM medium supplemented with 10% FBS, 10 mM HEPES, 100 U per ml penicillin and 10 μg per ml streptomycin, and incubated at 37°C in a humidified atmosphere containing 95% air/5% CO2.

### Animal tumor model

Female athymic nude mice (6-weeks-old) were purchased from Shanghai Laboratory Animal Centre (Chinese Academy of Sciences, Shanghai, China) and maintained in cage housing under specific pathogen-free conditions. HeLa cells were harvested from 6-well plates and resuspended in 0.2 ml of PBS at 5 × 10^7^ cells/ml. Cells were injected into tail vein of the mice to generate the lung metastasis model. Following injection, the mice (ten mice per group) were treated with 100 mg/kg ART or vehicle (sterile PBS) by subcutaneous injection, once per day. After 6 weeks, the mice were killed and the lungs were removed, fixed with 10% formaldehyde and sectioned. The weight of the lungs was measured, and visible tumors on the lung surface (more than 0.5 mm in diameter) were counted. All animals were euthanized by CO_2_ asphyxiation followed by cervical dislocation. All efforts were made to minimize animal suffering. This study was carried out in accordance with the guidelines of the Research Animal Care Committee of Nantong University of medicine (Taizhou, China) and was approved by the Committee on the Ethics of Animal Experiments of Nantong University of medicine.

### ELISA

Analysis of PGE_2_ production was performed using an ELISA kit according to manufacturer’s instructions. CaSki and HeLa cells were transfected with pcDNA3.1 or pcDNA3.1-HOTAIR for 24 h, then, treated with ART for another 24 h. The cell culture supernatants were collected in order to measure the concentration of PGE_2_. Production of PGE_2_ was normalized to protein concentrations.

### Real-time PCR assay

Total RNA of CaSki and HeLa cells were extracted using TRIzol reagent (Invitrogen, Grand Island, NY, USA) according to the manufacturer’s protocol. The expression of mRNA and LncRNA was quantified by real-time PCR using a LightCycler480 II Sequence Detection System (Roche, Basel, Switzerland). The specific primers were as follows: COX-2, 5’-CCGAGGTGTATGTATGAGTG-3’ (forward) and 5’-AACTGATGCGTGAAGTGCTG-3’ (reverse); HOTAIR, 5’-CAGT GGGGAACTCTGACTCG-3’ (forward) and 5’-GTGCCTGGTGCTCTCTTACC-3’ (reverse); CCAT2, 5’-CCACATCGCTCAGACACCAT-3’ (forward) and 5’-ACCA GGCGCCCAATACG-3’ (reverse); MALAT1, 5’-AAAGCAAGGTCTCCCCACAA G-3’ (forward) and 5’-GGTCTGTGCTAGATCAAAAGGCA-3’ (reverse); EBIC, 5’-GACTGAATGGACAAGTGGATCTTC3’ (forward) and 5’-GGAGTTCTTCTT GACCCTCTTGTAG-3’ (reverse); MEG3, 5’-CAGCCAAGCTTCTTGAAAGG-3’ (forward) and 5’-TTCCACGGAGTAGAGCGAGT-3’ (reverse); GAPDH, 5’-GGGA GCCAAAAGGGTCAT-3’ (forward); and 5’-GAGTCCTTCCACGATACCAA-3’ (reverse). GAPDH was used as an internal control.

### Western blot analysis

The total protein was isolated from CaSki and HeLa cells. Nuclear extracts were prepared from CaSki cells by using NE-PERTM Nuclear and Cytoplasmic Extraction Reagents (Pierce, Rockford, IL). Western blot analysis was performed as described [9]. Individual immunoblots were probed with a rabbit anti-COX-2 antibody (diluted 1:1000) and a mouse anti-β-Actin (diluted 1:3000).

### Plasmid construction

The HOTAIR expression plasmid (pcDNA3.1-HOTAIR) was constructed as previous report [17]. The sequences of primers of HOTAIR were as follow: 5’-CATGGA TCCACATTCTGCCCTGATTTCCGGAACC-3’ (forward) and 5’-ACTCTCGAGCC ACCACACACACACAACCTACAC-3’ (reverse). The sequences of primers COX-2 were as follow: 5’- TATAAGCTTCCCTCAGACAGCA AAGCCTA -3’ (forward) and 5’- CTAGTCTAGACTACAGGTTCAGTCGAACGTT CTTTTAG -3’ (reverse). The plasmids of pcDNA3.1-HOTAIR and pcDNA3.1-COX-2 were sequenced and confirmed to be correct.

### Small interfering RNA

Small interfering RNA specific for HOTAIR (si-HOTAIR) and control siRNA (si-NC) was synthesized (Ribobio, Guangzhou, China) and transfected using Lipofectamine 2000 in CaSki and HeLa cells. The sequences of siRNA were: si-HOTAIR1, 5′-AAAUCCAGAACCCUCUGACAUUUGC-3′, si-HOTAIR2, 5′-UU AAGUCUAGGAAUCAGCACGAAGC-3′ and si-HOTAIR3, 5′-CAUAUUAUAGA GUUGCU CUGUGCUG-3′.

### RNA immunoprecipitation (RIP)

RIP experiments were performed using the Magna RIP^™^ RNA-binding protein immunoprecipitation kit (Millipore, Bedford, MA, USA) according to the manufacturer’s protocol. The antibody for RIP assays of COX-2 was diluted 1:50. Co-precipitated RNAs were detected by Real-time PCR assay.

### RNA pull-down assay

HOTAIR and its antisense RNA were in vitro transcribed and biotin-labeled with the Biotin RNA Labeling Mix (Roche Diagnostics, Indianapolis, IN) and SP6 RNA polymerase (Roche), and purified with an RNeasy Mini Kit (Qiagen, Valencia, CA). One milligram of protein from CaSki cell extracts was mixed with 50 pmol of biotinylated RNA, incubated with streptavidin agarose beads (Invitrogen, Carlsbad, CA), and washed. The retrieved proteins were detected using Western blot analysis.

### Invasion and migration assay

For the migration assays, 1 × 10^5^ cells in serum-free media were placed into the upper chamber of a Transwell insert (8-μm pore size; Millipore). For the invasion assays, cells in serum-free medium were placed into the upper chamber of an insert coated with Matrigel (Sigma-Aldrich). Medium containing 10% FBS was added to the lower chamber. After incubation for 16 h, the cells remaining on the upper membrane were removed with cotton wool. Cells that had migrated or invaded through the membrane were fixed in methanol, stained with crystal violet (0.04% in water; 100 μl), counted using an inverted microscope and photographed.

### Statistical analysis

Statistical analyses were performed using SPSS 13.0 statistical analysis software and were performed using either an analysis of variance (ANOVA) or Student’s *t*-test. Data are expressed as mean ± standard deviation. *P* < 0.05 was considered to indicate a statistically significant difference.

## Results

### ART suppressed cervical cancer metastasis in vivo

**Fig 1:**
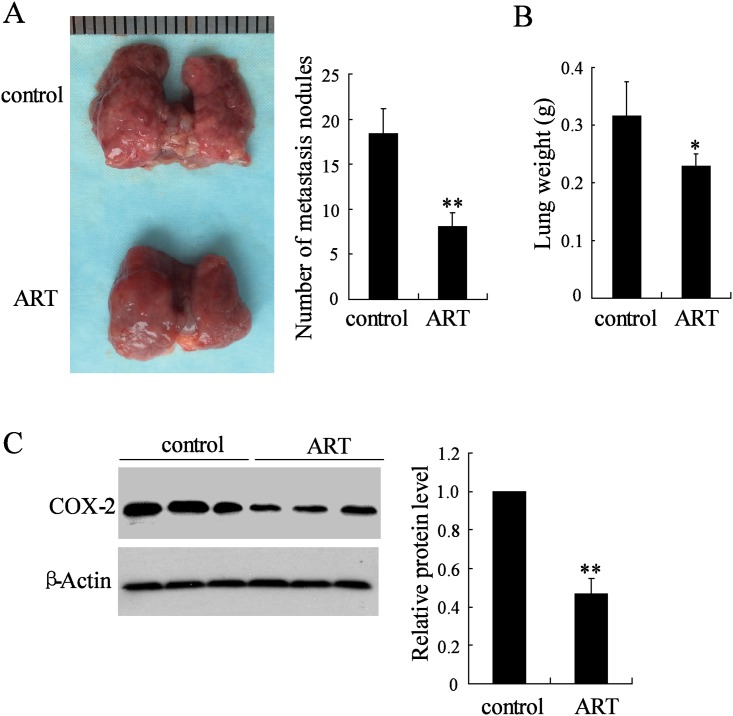
ART suppressed cervical cancer metastasis in vivo. HeLa cells were injected into nude mice via the tail vein. Following injection, the mice (ten mice per group) were treated with 100 mg/kg ART or vehicle (sterile PBS) by subcutaneous injection once per day. After 6 weeks, (A) the numbers of tumor nodules on lung surfaces, (B) the weight of the lungs and (C) COX-2 expression was measured. * P < 0.05, **P < 0.01, compared to control.

Our previous study showed that ART inhibited tumor growth in the cervical cancer mice [9]. To investigate the *in vivo* effects of ART on the metastasis of cervical cancer cells, we injected HeLa cells into nude mice via the tail vein. ART treatment significantly decreased the number of metastatic nodules when compared with the PBS-injected mice (Fig 1A). Mean lung weights for animals that received ART (0.23 ± 0.02g) were significantly lower than those from control animals (0.32 ± 0.06g, Fig 1B). In addition, we found that ART inhibited the expression of COX2 in the animal tumors in Fig 1C.

### ART inhibited HOTAIR expression in cervical cancer cells

**Fig 2:**
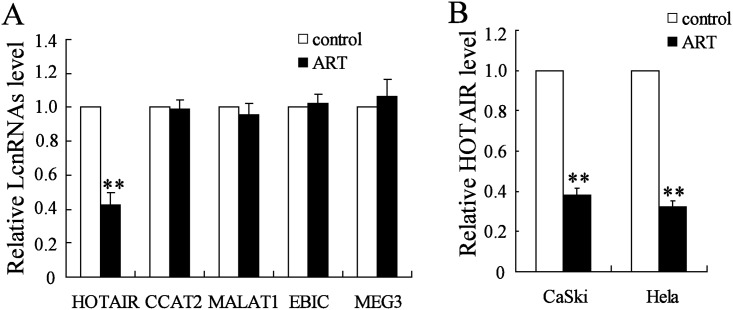
ART inhibited HOTAIR expression in cervical cancer cells. (A) The expression of HOTAIR, CCAT2, MALAT1, EBIC and MEG3 was measured in metastatic nodules from mice treated with ART or PBS. (B) The HOTAIR expression was measured in CaSki and Hela cells treated with or without ART. **P < 0.01, compared to control. **P < 0.01, compared to control.

LcnRNAs of HOTAIR, CCAT2, MALAT1, EBIC and MEG3 have been reported to be involvled in cervical cancer metastasis [18–21]. We measured the expression of these LcnRNAs in metastatic nodules from mice treated with ART or PBS. As shown in Fig 2A, ART significantly inhibited HOTAIR expression in metastatic nodules but had no effect on the expression of CCAT2, MALAT1, EBIC and MEG3. In addition, we also determined the HOTAIR expression in CaSki and Hela cells treated with ART. Our results showed that ART significantly decreased HOTAIR expression in cervical cancer cells (Fig 2B).

### Overexpression of HOTAIR reversed the effect of ART on cervical cancer cell migration and invasion

**Fig 3:**
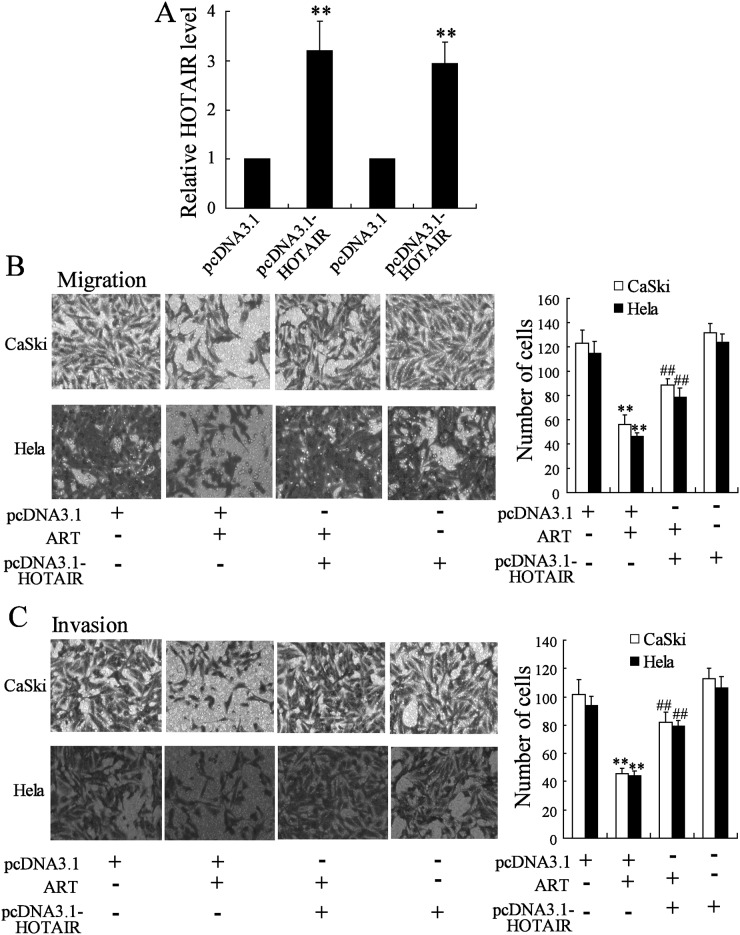
Overexpression of HOTAIR reversed the effect of ART on cervical cancer cell migration and invasion. (A) CaSki and Hela cells was transfected with pcDNA3.1 or pcDNA3.1-HOTAIR for 24 h, then, HOTAIR expression was measured. After transfected with pcDNA3.1 or pcDNA3.1-HOTAIR for 24 h, CaSki and Hela cells were treated with ART (50 mmol/l) for 24 h. Then, (B) transwell assays were performed and (C) PGE_2_ concentration was measured. **P < 0.01, compared to pcDNA3.1. ##P < 0.01, compared to pcDNA3.1 + ART.

To establish the role of HOTAIR in metastasis of cervical cancer cells, CaSki and Hela cells were transfected with pcDNA3.1-HOTAIR. As shown in Fig 3A, HOTAIR expression was significantly increased in cells transfected with pcDNA3.1-HOTAIR. ART caused a significant decrease in cervical cancer cell migration, which was obviously reduced by HOTAIR overexpression (Fig 3B). Similar results of cervical cancer cell invasion (Fig 3C).

### HOTAIR regulates COX-2 expression and PGE_2_ production in cervical cancer cells

**Fig 4:**
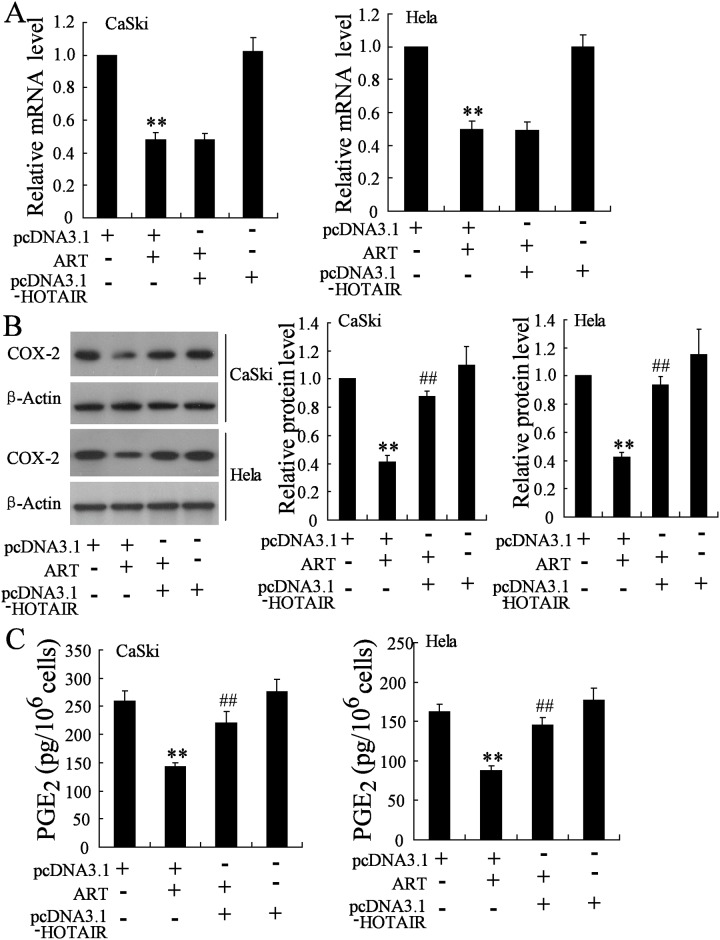
HOTAIR regulates COX-2 expression and PGE_2_ production in cervical cancer cells. After transfected with pcDNA3.1 or pcDNA3.1-HOTAIR for 24 h, CaSki and Hela cells were treated with ART (50 mmol/l) for 24 h. Then, (A) COX-2 mRNA level, (B) COX-2 protein level and (C) PGE_2_ concentration were measured. **P < 0.01, compared to pcDNA3.1. ##P < 0.01, compared to pcDNA3.1 + ART.

As shown in Fig 4A and 4B, HOTAIR overexpression abolished ART-induced the decrease of COX-2 protein level but had no effect on COX-2 mRNA in cervical cancer cells. ART treatment resulted in a significant decrease in the concentrations of PGE_2_ in the supernatants of CaSki and Hela cells, which were reversed by HOTAIR overexpression (Fig 4C).

**Fig 5:**
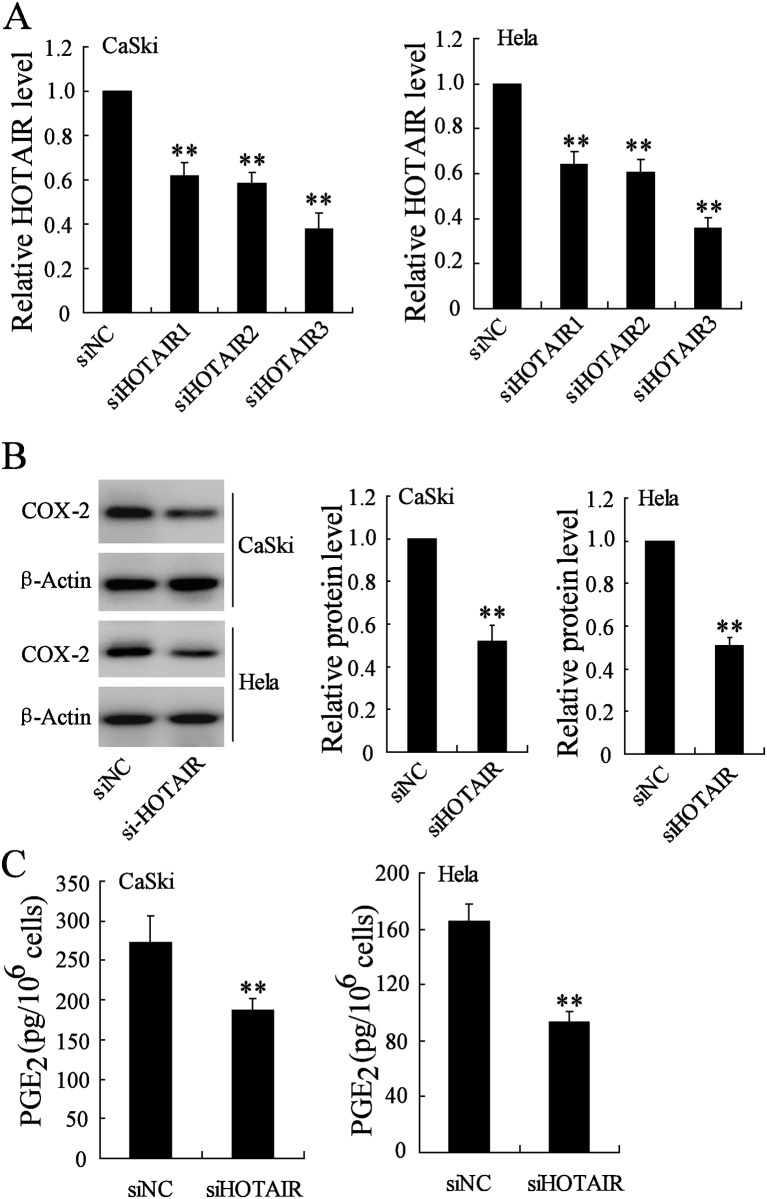
Downregulation of HOTAIR inhibits COX-2 expression and PGE_2_ production in cervical cancer cells. **(A)** CaSki and Hela cells were transfected with 3 HOTAIR-specific siRNAs (siHOTAIR1, siHOTAIR2 and siHOTAIR3) for 24 h, then, HOTAIR expression was measured. CaSki and Hela cells were transfected with siHOTAIR3 (si-HOTAIR) for 24 h, then, (B) COX-2 protein level and (C) PGE_2_ production were measured. **P < 0.01, compared to siNC.

To further investigate the effect of HOTAIR on COX-2 expression and activity, the siRNA of HOTAIR has been transfected in CaSki and Hela cells. The knockdown efficiency of the 3 HOTAIR-specific siRNAs (siHOTAIR1, siHOTAIR2 and siHOTAIR3) was evaluated, and siHOTAIR3 was found to have highest silencing efficiency (Fig 5A). Therefore, siHOTAIR3 was selected for use in the subsequent experiments. Depletion of HOTAIR led to a decrease of COX-2 protein level and PGE_2_ production (Fig 5B and 5C). These results indicated that HOTAIR positively regulated COX-2 expression and catalytic activity in cervical cancer cells.

### HOTAIR interacts with COX-2 in cervical cancer cells

**Fig 6:**
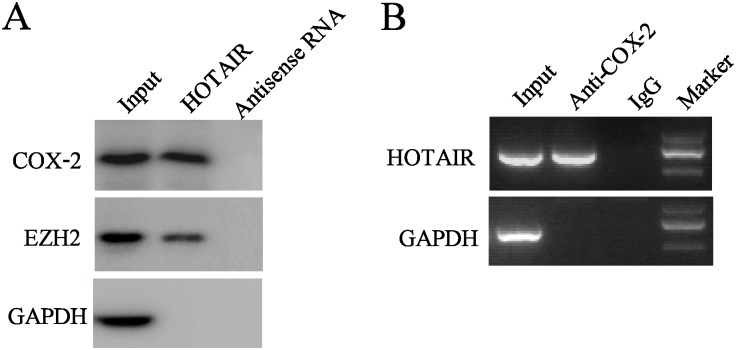
HOTAIR interacts with COX-2 in cervical cancer cells. (A) Biotin-labeled HOTAIR or antisense RNA was incubated with nucleoprotein from CaSki cell extracts, and the protein of COX-2 and EZH2 was assayed by Western blot. A non-specific protein (GAPDH) was used as the control. (B) RIP experiments were performed in CaSki cells using an COX-2 antibody or non-specific IgG, and specific primers were used to detect HOTAIR and GAPDH.

Previous studies have demonstrated that HOTAIR could directly interact with target proteins and increase their stability [22]. To investigate whether HOTAIR regulated COX-2 expression in such a manner, biotin-labeled HOTAIR was transfected into cervical cancer cells. Then, we performed RIP and RNA pull-down assays to identify whether COX-2 was associated with HOTAIR. As shown in Fig 6A, HOTAIR was selectively interacting with COX-2. COX-2 expression was determined by Western blot analysis in RNA pull-down assays. We also observed that HOTAIR could interact with EZH2, which was consistent with previous report [23]. Moreover, HOTAIR enrichment in RIP using COX-2 antibody in nuclear extracts from CaSki cells (Fig 6B).

### COX-2 is required for the effect of HOTAIR on cervical cancer cell migration and invasion

**Fig 7:**
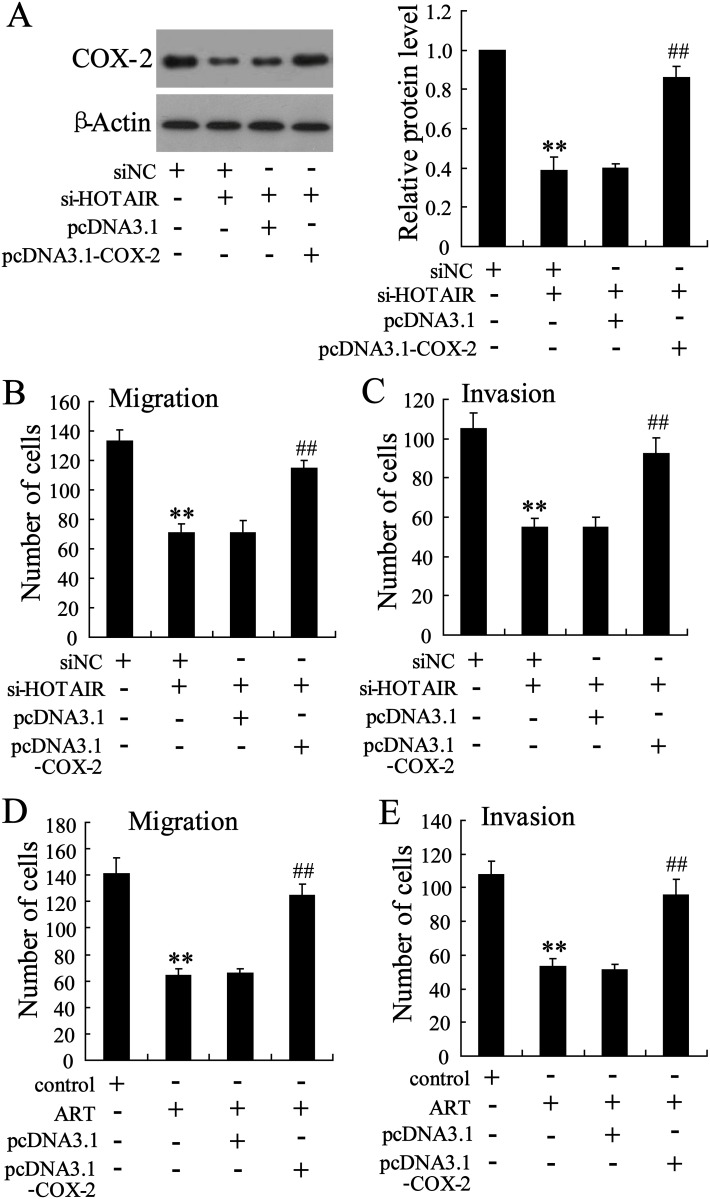
COX-2 is required for the effect of HOTAIR on cervical cancer cell migration and invasion. CaSki cells were transfected with si-HOTAIR and pcDNA3.1-COX-2 for 48 h, then (A) COX-2 protein was measured and (B and C) transwell assays were performed. **P < 0.01, compared to siNC. ##P < 0.01, compared to si-HOTAIR + pcDNA3.1. After transfected with pcDNA3.1 or pcDNA3.1-COX-2 for 24 h, CaSki cells were treated with ART (50 mmol/l) for 24 h. Then, (D and E) transwell assays were performed. **P < 0.01, compared to control. ##P < 0.01, compared to pcDNA3.1 + ART.

Kim et al reported that HOTAIR knockdown reduced cervical cancer cell migration and invasion [17]. To explore whether COX-2 mediated the effect of HOTAIR on cervical cancer cell migration and invasion, pcDNA3.1-COX-2 was transfected in CaSki cells. HOTAIR knockdown in Hela cells decreased COX-2 protein level, which was reversed by transfection of pcDNA3.1-COX-2 (Fig 7A). In addition, knockdown of HOTAIR inhibited CaSki cell migration and invasion, which was reversed by overexpression of COX-2 (Fig 7B and 7C). We also found that overexpression of COX2 reversed the effect of ART on cervical cancer cell migration and invasion (Fig 7D and 7E).

## Discussion

ART is derived from a common traditional Chinese medicine and has anti-cancer activities. Previously, we have reported that ART has an anti-immunosuppressive effect on cervical cancer [9]. The present study revealed the molecular mechanism by which ART exerted an anti-metastatic effect on cervical cancer cells. We found that ART inhibited cervical cancer metastasis *in vivo* and *in vitro*. It is important to note that low dosage of ART did not decrease the viability of CaSki and Hela cells [9]. Thus, the decrease of invasion and migration upon ART treatment could not be the result of decrease of the cell number.

Growing evidence indicates that LcnRNAs play key roles in cervical cancer metastasis [17–21]. The present study demonstrated that HOTAIR expression was obviously decreased in cervical cancer cells treated with ART. Given HOTAIR promoted metastasis of cervical cancer [17], we further speculated donwregulation of HOTAIR expression mediated an anti-metastatic effect of ART on cervical cancer. This speculation was supported by the observation that HOTAIR overexpression partially abolished the effect of ART on cervical cancer cell migration and invasion. These findings established a novel mechanism of anti-metastasis of ART.

The present data additionally demonstrated that HOTAIR positively regulated COX-2 expression and catalytic activity in cervical cancer cells. HOTAIR overexpression increased COX-2 expression and PGE_2_ production in cervical cancer cells treated with ART, while HOTAIR knockdown decreased COX-2 expression and PGE_2_ production. Besides, HOTAIR had no effect on COX-2 mRNA level. Therefore, HOTAIR regulated COX-2 expression at post-transcriptional level. Increasing evidence has confirmed that HOTAIR can directly interact with proteins and change the activity of these proteins [22]. Our results showed that HOTAIR bound nuclear COX-2 protein and increased COX-2 protein level. It has been reported that HOTAIR is implicated in protein ubiquination [22, 24]. We therefore hypothesized that HOTAIR regulating COX-2 expression via the ubiquitin proteasome pathway. However, whether other molecules are involved in interaction of HOTAIR and COX-2 requires further study.

COX-2 contributes to tumor metastasis and acts as the key molecular to treat cervical cancer [25]. Our previous study demonstrated that ART exerted immunosuppressive effect by inhibiting COX-2 expression and catalytic activity [9]. In the current study, we found that ART inhibited the expression of COX2 in the animal tumors. COX-2 overexpression reversed the effect of ART on cervical cancer cell migration and invasion. More importantly, overexpression of COX-2 effectively abolished the decrease of cervical cancer cell migration and invasion by HOTAIR knockdown. This finding indicated that the effect of HOTAIR on cell migration and invasion was dependent on COX-2.

In conclusion, this study demonstrated that ART effectively inhibited HOTAIR expression, leading to reduction of COX-2 expression and catalytic activity, and finally to the decrease of cervical cancer cell invasion and migration. This process may be one of the molecular mechanisms of anti-metastasis of ART.
